# Numerical study on the lubrication performance of compression ring-cylinder liner system with spherical dimples

**DOI:** 10.1371/journal.pone.0181574

**Published:** 2017-07-21

**Authors:** Cheng Liu, Yan-Jun Lu, Yong-Fang Zhang, Sha Li, Norbert Müller

**Affiliations:** 1 School of Mechanical and Precision Instrument Engineering, Xi’an University of Technology, Xi’an, China; 2 State Key Laboratory for Strength and Vibration of Mechanical Structures, Xi’an Jiaotong University, Xi’an, China; 3 School of Printing, Packaging Engineering and Digital Media Technology, Xi’an University of Technology, Xi’an, China; 4 College of Engineering, Michigan State University, East Lansing, Michigan, United States of America; Lanzhou University of Technology, CHINA

## Abstract

The effects of surface texture on the lubrication performance of a compression ring-cylinder liner system are studied in this paper. By considering the surface roughness of the compression ring and cylinder liner, a mixed lubrication model is presented to investigate the tribological behaviors of a barrel-shaped compression ring-cylinder liner system with spherical dimples on the liner. In order to determine the rupture and reformulation positions of fluid film accurately, the Jacoboson-Floberg-Olsson (JFO) cavitation boundary condition is applied to the mixed lubrication model for ensuring the mass-conservative law. On this basis, the minimum oil film thickness and average friction forces in the compression ring-cylinder liner system are investigated under the engine-like conditions by changing the dimple area density, radius, and depth. The wear load, average friction forces, and power loss of the compression ring-cylinder liner system with and without dimples are also compared for different compression ring face profiles. The results show that the spherical dimples can produce a larger reduction of friction in mixed lubrication region, and reduce power loss significantly in the middle of the strokes. In addition, higher reduction percentages of average friction forces and wear are obtained for smaller crown height or larger axial width.

## Introduction

In order to save energy and improve efficiency of internal combustion engines (ICEs), more attention has been paid to the friction reduction of piston ring-cylinder liner system because friction loss contributes significantly to the power loss of ICEs [1]. Recently, with the development of surface processing technology, surface texturing has been applied to reduce friction [2–4] and increase load-carrying capacity [5–7] of modern machine components due to its trapping capability of wear debris, second lubrication and micro-hydrodynamic effects [8]. Texturing well-designed micro dimples/grooves on the piston ring or cylinder liner has been recognized as a promising way to reduce friction, wear, and oil consumption of ICEs [9, 10].

Because dimples/grooves geometry, shape, and distribution pattern have important influence on the lubrication performance, their effects on the lubrication performance of a piston ring-cylinder liner system were extensively studied in two decades to decrease the friction and wear loss maximally. These studies are mainly divided into two categories: one category focuses on the dimple/groove textured on piston ring, another focuses on the dimple/groove textured on cylinder liner. For the dimple/groove textured on piston ring, Etsion and his co-workers have carried out some significant research works on the improvement of lubrication performance in a piston ring-cylinder liner system [2,11,12]. They suggested that the partial dimples should be textured on the flat piston ring to obtain the minimum friction and oil consumption. Gadeschi et al. investigated the friction force and load-carrying capacity of a barrel-shaped piston ring with partial laser dimples [13]. The dimple depth, density, and distribution pattern were optimized to minimize the friction coefficient for the ring with various curvatures. Zavos et al. investigated the friction force of a flat piston ring with rectangular or spherical dimples under various operating conditions [14]. A higher friction reduction was obtained for the rectangular dimples with density of 61%, depth of 4 μm, aspect ratio of 20, and relative depth of 0.004. Shen et al. evaluated the frictional performance of a piston ring-cylinder liner system with micro pockets on the ring [15]. A significant friction reduction was achieved for the pockets with area density of 25% and depth of 5 μm. Usman et al. investigated the tribological characteristics of a barrel-shaped compression ring with transverse grooves, axial grooves, and dimples under warm-engine conditions [16]. The minimum friction loss was obtained when the transverse grooves were textured at the edges of the ring.

Texturing dimples/grooves on cylinder liner surface for friction and wear reduction is another research focus because of smaller and more even wear loss of cylinder liner, which can preserve shape and size of dimples/grooves for long life cycle [17]. Yin et al. studied the lubrication performance of a flat compression ring-cylinder liner system with micro spherical dimples on the liner [18]. In their work, the maximum reduction in average friction force was obtained by choosing the suitable radius, area density, and depth of dimples. Zhou et al. textured uniform shape dimples with different area densities and depth over diameter ratios on a cylinder liner to obtain higher load-carrying capacity and oil film thickness [19]. By texturing grooves with suitable shape and size on the cylinder liner, Mohamad et al. obtained lower friction and higher hydrodynamic pressure [20,21]. These studies are mainly focused on the flat piston ring-cylinder liner system with dimples/grooves on the liner. However, compared with the flat piston ring, the non-conformal barrel-shaped piston ring is more prevalent in ICEs, especially for the top compression ring [17,22]. Zhan et al. studied the effect of liner dimple distribution angle on the wear characteristics of a barrel-shaped piston ring-cylinder liner system experimentally [23]. The wear value was decreased by 78.6% when the optimal dimple distribution angle was chosen. Gu et al. investigated the wear and friction loss of a barrel-shaped compression ring-cylinder liner system with full or partial groove on the ring or liner [24]. They found that the fully textured liner has lower wear and friction loss in some conditions. Morris et al. studied the effect of the depth of chevrons on the friction reduction of a barrel-shaped compression ring-cylinder liner system at top dead center [25]. A marginal reduction in friction was obtained for the chevrons with shallow depth. In these numerical studies, the rupture of oil film was addressed by Reynolds cavitation boundary condition, which is not mass conservative and the oil film reformulation is not taken into consideration.

During the operation of barrel-shaped piston ring-cylinder liner system, the cavitation phenomenon of oil film occurs when the pressure is lower than a given cavitation pressure, the oil film will experience rupture and reformulation multiple times [26,27]. It is generally accepted that a mass conservative cavitation boundary condition is required for an accurate performance prediction [26–28]. The JFO cavitation boundary condition can address the oil film rupture and reformulation simultaneously, and meets the requirement of mass conservative. It has been experimentally validated and widely accepted in the study of textured surface [29]. Based on mass conservative JFO cavitation boundary condition, Checo et al. investigated the lubrication performance of a barrel-shaped compression ring-cylinder liner system with liner dimples in hydrodynamic lubrication regime [30]. In their work, the velocity and load of compression ring were assumed to be constant. However, in practice, the velocity and load of compression ring are time-varying, and the compression ring-cylinder liner system is often under mixed or boundary lubrication regimes, particularly at the dead centers [25,28]. It seems logical to consider the role of surface roughness and engine-like working conditions (i.e., time-varying velocity and load) in the lubrication analysis of the textured compression ring-cylinder liner system.

On the basis of the aforementioned studies, the Reynolds cavitation boundary condition and the ideal working conditions (i.e., constant velocity and load) are usually adopted in the performance analysis of barrel-shaped compression ring-cylinder liner system with dimples/grooves on the liner. In this paper, a mixed lubrication model coupled with mass conservative JFO cavitation boundary condition is presented to investigate the lubrication performance of a barrel-shaped compression ring-cylinder liner system with spherical dimples on the liner. By choosing the suitable parameters of the radius, area density, and depth of the dimples, an insight is provided into the frictional characteristics of the barrel-shaped compression ring under the engine-like working conditions.

## Method

### Geometry model

**Fig 1:**
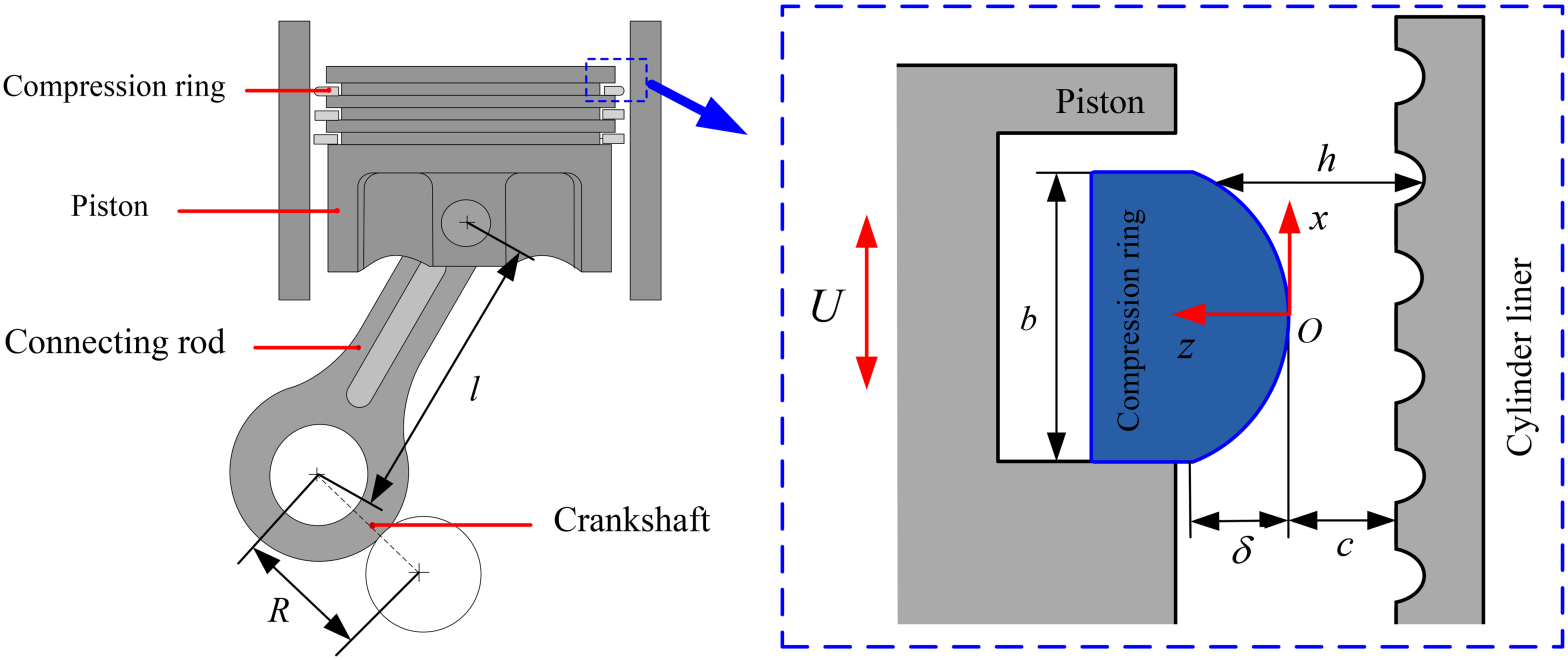
Schematic diagram of a barrel-shaped compression ring-cylinder liner system.

Fig 1 shows the schematic diagram of a barrel-shaped compression ring-cylinder liner system. The barrel-shaped compression ring moves up and down in the cylinder liner to provide a seal between the combustion chamber and the crankcase. The time-varying velocity of compression ring can be expressed as [23]:

$$U\approx R\omega \text{sin}(\omega t)+\frac{R^{2}\omega }{2l}\text{sin}(2\omega t)$$ (1)

where *R* is the radius of crankshaft, *ω* is the angular velocity of crankshaft, *l* is the length of connecting rod.

**Fig 2:**
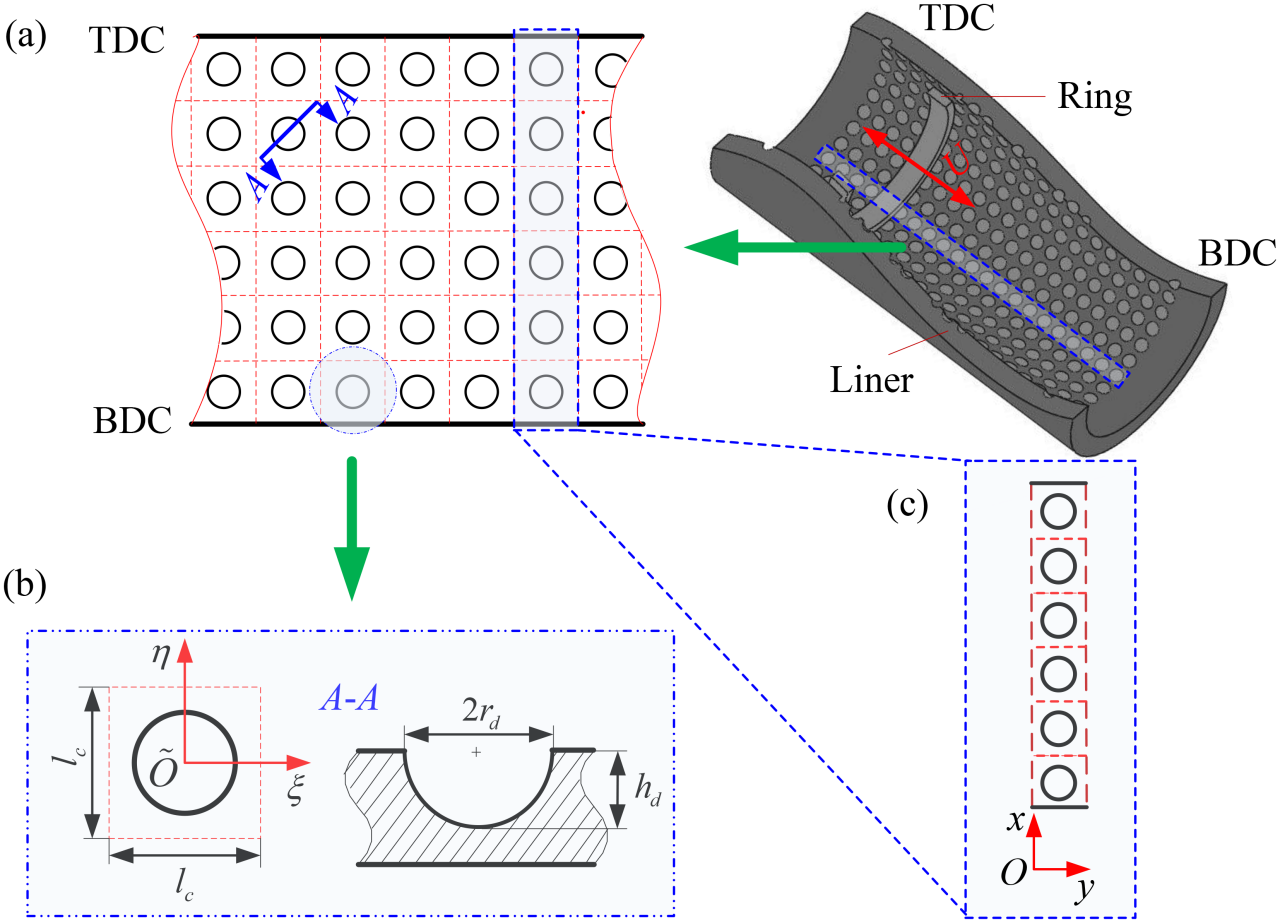
Schematic diagram of the cylinder liner with micro spherical dimples.

As micro spherical dimples have the advantage of easier fabrication, the frictional characteristics of a barrel-shaped compression ring-cylinder liner system with micro spherical dimples on the liner are investigated in this paper. Fig 2 shows the schematic diagram of the cylinder liner with micro spherical dimples. In order to display the geometry of cylinder liner with micro spherical dimples clearly, a half of cylinder liner is presented. In Fig 2, the micro spherical dimples are uniformly textured on the inner surface of the cylinder liner from top dead center to bottom dead center. *r*_*d*_ is the dimple radius, *h*_*d*_ is the maximum depth of dimple, and *ξÕη* is the local coordinate system located in the dimple center. In order to facilitate the analysis of friction characteristics, the dimple with area density *s* is supposed to be located in the center of a imaginary square cell of sizes *l*_*c*_×*l*_*c*_. The dimple area density *s* is defined as [18]:

$$s=\frac{\pi r_{d}^{2}}{l_{c}^{2}}$$ (2)

### Governing equation

For the barrel-shaped compression ring-cylinder liner system with micro spherical dimples, the oil film may rupture when the pressure is lower than a given cavitation pressure. The lubrication region can be divided into the full film region and cavitation region. In the full film region, the oil film pressure distribution considering the surface roughness between the compression ring and the cylinder liner can be described by the following average Reynolds equation [31–33]:

$$\frac{\partial }{\partial x}(\varphi_{x}\frac{\rho h^{3}}{\mu }\frac{\partial p}{\partial x})+\frac{\partial }{\partial y}(\varphi_{y}\frac{\rho h^{3}}{\mu }\frac{\partial p}{\partial y})=6U\varphi_{c}\rho \frac{\partial h}{\partial x}+6U\sigma \frac{\partial (\varphi_{s}\rho )}{\partial x}+12\varphi_{c}\rho \frac{\partial h}{\partial t}$$ (3)

where *x* is the axial coordinate, *y* is the circumferential coordinate, *p* is the pressure of oil film, *μ* is the oil viscosity, *h* is the nominal oil film thickness, *ρ* is the oil density, *φ*_*x*_ and *φ*_*y*_ are the pressure flow factors, *φ*_*s*_ is the shear flow factor, *φ*_*c*_ is the contact factor, *σ* is the comprehensive surface roughness, and *t* is the time.

In the cavitation region, the oil film pressure equals to the constant cavitation pressure *p*_*c*_, and the average Reynolds equation can be rewritten as [33]:

$$U\varphi_{c}\rho_{c}\frac{\partial h}{\partial x}+U\sigma \frac{\partial (\varphi_{s}\rho_{c})}{\partial x}+2\varphi_{c}\rho_{c}\frac{\partial h}{\partial t}=0$$ (4)

where *ρ*_*c*_ is the density of oil-gas mixture in the cavitation region.

The mass conservative JFO cavitation boundary conditions are used to determine the oil film rupture and reformulation boundary positions because the JFO cavitation boundary condition can capture the features of cavitation more accurately [29]. The mass conservative JFO boundary conditions are given as follow [30]:

$$\{\begin{array}{ll} \frac{\partial p}{\partial n}=0 & \left(\text{rupture boundary}\right)\\ \frac{h^{2}}{12\mu }\frac{\partial p}{\partial n}=U\frac{\rho -\rho_{c}}{2\rho } & \left(\text{reformation boundary}\right) \end{array}$$ (5)

where *n* is the normal direction of the rupture or reformation boundary.

The governing equations with JFO cavitation boundary conditions can be expressed as a universal equation by the Payvar-Salant method in the full film and cavitation regions [34]. The universal average Reynolds equation is expressed in the following form:

$$\begin{array}{l} \frac{\partial }{\partial x}(\varphi_{x}\frac{p_{a}h^{3}}{\mu }\frac{\partial Dr}{\partial x})+\frac{\partial }{\partial y}(\varphi_{y}\frac{p_{a}h^{3}}{\mu }\frac{\partial Dr}{\partial y})=6U\varphi_{c}[1+(1-D)r]\frac{\partial h}{\partial x}+\\ 6U\sigma \frac{\partial \left\{[1+(1-D)r]\varphi_{s}\right\}}{\partial x}+12[1+(1-D)r]\varphi_{c}\frac{\partial h}{\partial t} \end{array}$$ (6)

where *p*_*a*_ is the ambient pressure. The universal variable *r* and the cavitation index *D* are defined as follow:

$$\{\begin{array}{ll} r=\frac{p-p_{c}}{p_{a}} & \left(\text{full film region}\right)\\ r=\frac{\rho_{c}-\rho }{\rho } & \left(\text{cavitation region}\right) \end{array}$$ (7)

$$\{\begin{array}{ll} D=1 & r\geq 0\\ D=0 & r<0 \end{array}$$ (8)

### Film thickness equation

In order to investigate the lubrication performance of compression ring-cylinder liner system with spherical dimples on the liner, the oil film thickness between the compression ring and the cylinder liner should be well characterized. When the cylinder liner is textured with micro spherical dimples, the moving dimples proposed by Checo et al. [30] should be taken into consideration. In this case, the oil film thickness is time-varying, and it can be expressed as:

$$h(x,y)=\{\begin{array}{ll} h_{r}(x)+c(t)+h_{l}(x,y,t) & (x,\;y)\in \Omega \\ h_{r}(x)+c(t)\; & (x,\;y)\notin \Omega \end{array}$$ (9)

where *c*(*t*) is the minimum oil film thickness between the compression ring and the cylinder liner, Ω is the dimple area in cell, *h*_*r*_(*x*) is the face profile of the barrel-shaped compression ring, and *h*_*l*_(*x*,*y*,*t*) is the oil film thickness variation due to the dimple textured on the cylinder liner. The expression of *h*_*r*_(*x*) [10] and *h*_*l*_(*x*,*y*,*t*) [2] can be written as:

$$h_{r}(x)=4\frac{\delta x^{2}}{b^{2}}$$ (10)

$$h_{l}(x,\;y,\;t)=\sqrt{{\left(\frac{r_{d}^{2}+h_{d}^{2}}{2h_{d}}\right)}^{2}-(\eta^{2}+\xi^{2})}-\frac{r_{d}^{2}-h_{d}^{2}}{2h_{d}}$$ (11)

where *δ* is the crown height of the compression ring, and *b* is the axial width of the compression ring.

### Motion equation of compression ring

**Fig 3:**
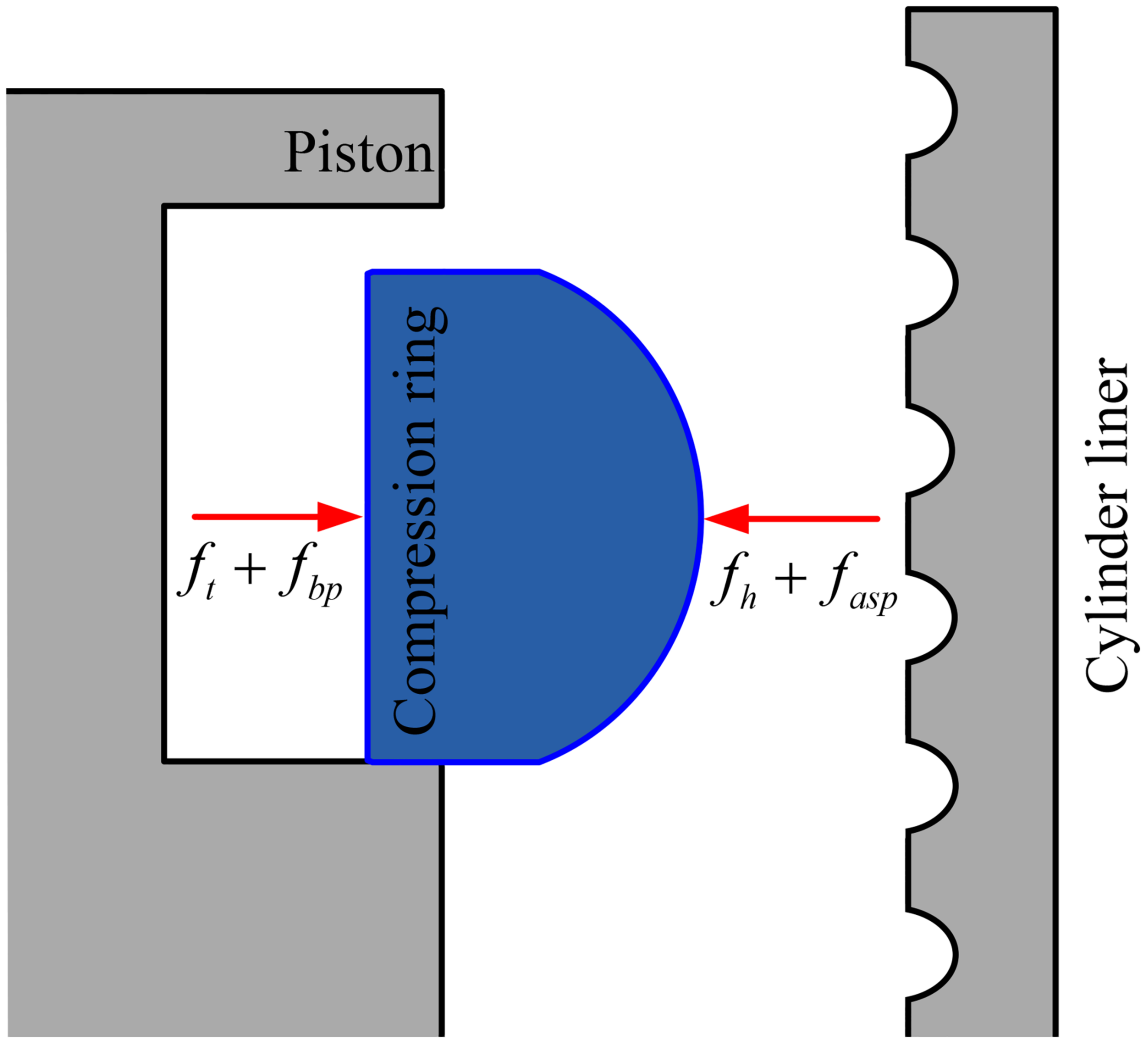
Radial forces acting on the compression ring.

Fig 3 shows the forces acting on the compression ring. In Fig 3, *f*_*t*_ is the tension force of the compression ring. *f*_*bp*_ is the backpressure of the compression ring. *f*_*h*_ is the oil film force, and *f*_*asp*_ is the asperity contact force. The oil film force and asperity contact force can be calculated by integrating the oil film hydrodynamic pressure *p* and the asperity contact pressure *p*_*asp*_, respectively.

During the running of the compression ring, the hydrodynamic pressure and the backpressure acting on the compression ring change with the change of crankshaft angles due to the time-varying velocity and cylinder pressure. Therefore, motion equation of the compression ring and universal average Reynolds equation should be solved simultaneously to obtain the time-varying minimum oil film thickness *c*(*t*) and the friction characteristics. The motion equation of the compression ring is given by:

$$m\frac{d^{2}c}{dt^{2}}=f_{h}+f_{asp}-f_{bp}-f_{t}$$ (12)

where *m* is the mass of the compression ring.

### Boundary conditions

**Fig 4:**
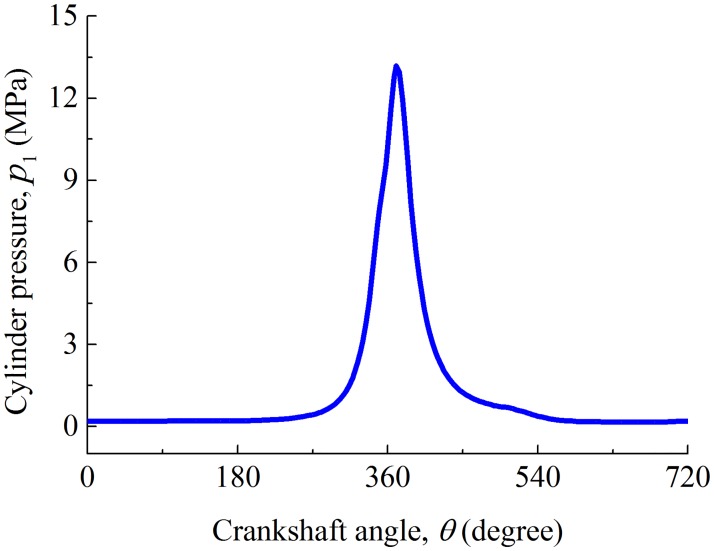
Cylinder pressure versus crankshaft angle.

The pressure boundary conditions in the axial direction can be expressed as [20]:

$$\{\begin{array}{l} \begin{array}{l} p{|}_{x=\frac{b}{2}}=p_{1}\\ p{|}_{x=-\frac{b}{2}}=p_{2} \end{array} \end{array}$$ (13)

where *p*_1_ is the cylinder pressure and *p*_2_ is the gas pressure under the compression ring. In practice, the cylinder pressure varies with time, and the gas pressure under the compression ring is almost equal to ambient pressure. In order to evaluate the friction characteristics accurately, the time-varying cylinder pressure is considered in this paper, as shown in Fig 4.

The dimples are uniformly distributed on the cylinder liner surface, the pressure distribution is periodic, the period equals the imaginary square cell size *l*_*c*_ in the circumferential direction [2,11]. Due to the periodic pressure distribution, one circumferential dimples column (with *y* varying from 0 to *l*_*c*_) is considered, as shown in Fig 2(c). Therefore, the following boundary conditions of pressure should be satisfied at *y* = 0 and *y* = *l*_*c*_ [35].

$$\{\begin{array}{l} \frac{\partial p}{\partial y}{|}_{y=0}=\frac{\partial p}{\partial y}{|}_{y=l_{c}}=0\\ p{|}_{y=0}=p{|}_{y=l_{c}} \end{array}$$ (14)

### Asperity contact model

At top dead center or bottom dead center, the compression ring-cylinder liner system is usually operating in the mixed lubrication regime, the asperity contact between the compression ring and the cylinder liner is inevitable. In order to investigate the asperity contact behaviors, the well-known Greenwood and Tripp’s model is employed in the present study. The asperity contact pressure *p*_*asp*_ and the asperity contact area *A*_*c*_ can be written as [36,37]:

$$p_{asp}=\frac{16\sqrt{2}}{15}\pi {\left(\kappa \beta \sigma \right)}^{2}E^{\prime }\sqrt{\frac{\sigma }{\beta }}F_{2.5}(\lambda )$$ (15)

$$A_{c}=\pi^{2}{\left(\kappa \beta \sigma \right)}^{2}AF_{2}(\lambda )$$ (16)

where *E'* is the equivalent elastic modulus, *κ* is the density of asperity, *β* is the curvature radius of asperity, *λ* is the ratio of the oil film thickness *h* to the comprehensive surface roughness *σ*, *A* is the apparent contact area. *F*_*j*_(*λ*) is a statistic function for modeling the roughness surfaces with Gaussian distributed asperities. It can be expressed as follow [37]:

$$F_{j}(\lambda )=\frac{1}{2\pi }\int_{\lambda }^{\infty }{\left(\chi -\lambda \right)}^{j}e^{-0.5\chi^{2}}d\chi$$ (17)

where *χ* is the correlation parameter.

### Performance parameters

The total friction force between the compression ring and the cylinder liner consists of hydrodynamic friction force and boundary friction force:

$$f=f_{v}+f_{b}$$ (18)

The hydrodynamic friction force *f*_*v*_ caused by the viscous shearing force of oil film can be calculated by [10,37]:

$$f_{v}=\underset{\Omega_{1}}{\iint }\{-\frac{\mu U}{h}(\varphi_{f}+\varphi_{fs})+\frac{h}{2}\frac{\partial p}{\partial x}\varphi_{fp}\}\text{d}\Omega_{1}$$ (19)

where *φ*_*f*_, *φ*_*fs*_ and *φ*_*fp*_ are the friction-induced flow factors, and Ω_1_ is the area of fluid film.

The boundary friction force *f*_*b*_ caused by the asperity contact can be calculated by [38]:

$$f_{b}=\tau_{0}A_{c}+\alpha_{0}f_{asp}$$ (20)

where *τ*_0_ is the shear stress constant and *α*_0_ is the boundary friction coefficient.

Notice that since the friction forces vary with crankshaft angle *θ*, presenting the results in forms of average friction forces makes logical sense. The average total friction force, average hydrodynamic friction force, and average boundary friction force over one complete engine cycle can be written as [39]:

$$F=\frac{1}{\theta_{0}}\int_{0}^{\theta_{0}}\left|f(\theta )\right|d\theta$$ (21)

$$F_{v}=\frac{1}{\theta_{0}}\int_{0}^{\theta_{0}}\left|f_{v}(\theta )\right|d\theta$$ (22)

$$F_{b}=\frac{1}{\theta_{0}}\int_{0}^{\theta_{0}}\left|f_{b}(\theta )\right|d\theta$$ (23)

where *θ*_0_ is 360° for a two-stroke ICE or 720° for a four-stroke ICE.

In order to understand the effect of micro dimples on the wear of compression ring-cylinder liner system, the wear load on the compression ring defined by Gulwadi [40] is calculated. The wear load can be expressed as [24,40]:

$$W_{load}=\frac{1}{\theta_{0}}\int_{0}^{\theta_{0}}p_{asp}\left|U\right|d\theta$$ (24)

The power loss of compression ring-cylinder liner system can be evaluated as [37]:

$$P_{loss}=\left|fU\right|$$ (25)

## Results and discussion

In order to provide insight into the lubrication performance of a barrel-shaped compression ring-cylinder liner system with micro spherical dimples on the liner, the minimum oil film thickness, friction force, wear load, and power loss are calculated by the proposed model. Table 1 shows the parameters of the compression ring-cylinder liner system.

**Table 1:** Parameters of the compression ring-cylinder liner system.

Parameter	Value
**Rotation speed of crankshaft, *ω***	146.5 rad·s^-1^
**Radius of crankshaft, *R***	0.05 m
**Diameter of cylinder liner, *d***	0.095 m
**length of connecting rod, *l***	0.155 m
**Surface roughness of ring, *σ***_**1**_	0.4×10^−6^ m
**Surface roughness of liner, *σ***_**2**_	0.4 ×10^−6^ m
**Equivalent elastic modulus, *E*'**	65.9×10^9^ Pa
**Tension force of compression ring, *f***_***t***_	20 N
**Crown height of compression ring, *δ***	1, 3, 5, 9 μm
**Axial width of compression ring, *b***	1.0, 1.5, 2.0 mm
**Oil viscosity, *μ***	0.03 Pa·s
**Shear stress constant, *τ***_**0**_	2 ×10^6^ Pa
**Boundary friction coefficient, *α***_**0**_	0.12
**Roughness parameters, *κβσ***	0.04
**Asperity gradient, *σ*/*β***	0.001

### Validation

**Fig 5:**
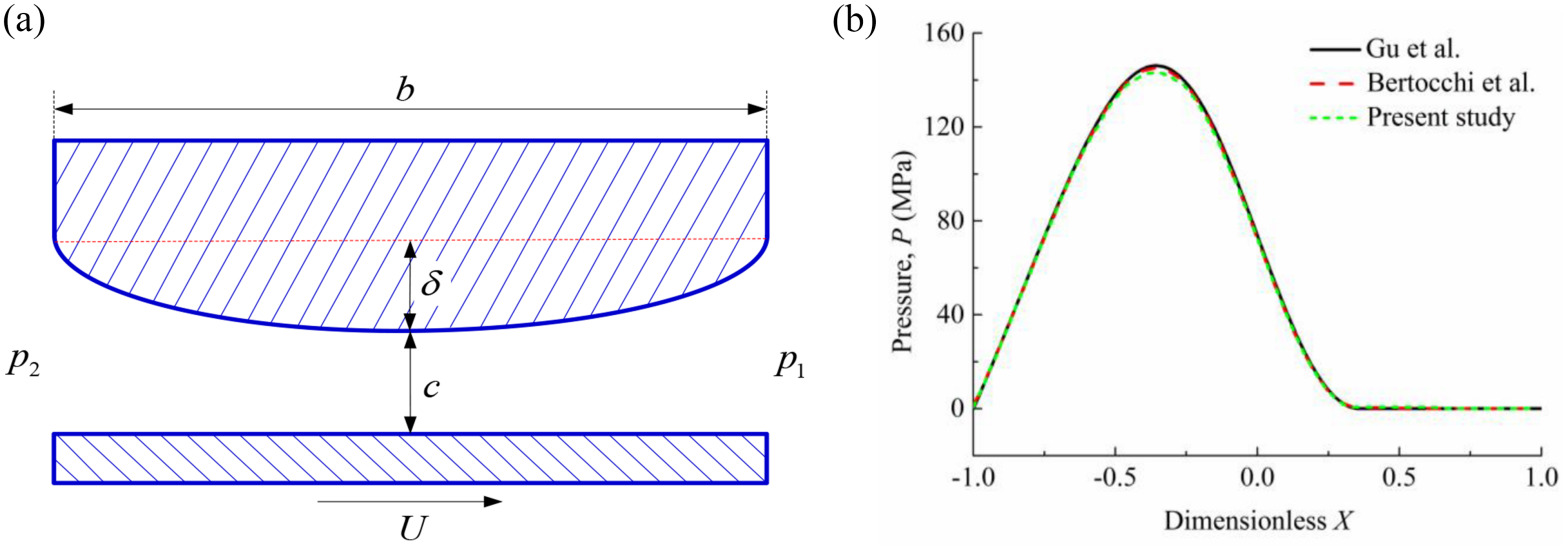
Model validation. **(a)** simulation conditions. **(b)** oil film pressures obtained by Gu et al.’s [28], Bertocchi et al. ‘s [41] and present model.

Before studying the effects of spherical dimple on the lubrication performance of compression ring-cylinder liner system, it is necessary to verify the model used in our study. The validation has been made using Bertocchi et al.’s [41], Gu et al.’s [28], and our models. Fig 5(a) shows the related parameters. In Fig 5(a), width *b* = 0.0762 m, transient velocity *U* = 4.57 m/s, inlet pressure *p*_1_ = 0 Pa, outlet pressure *p*_2_ = 3.36414×10^5^ Pa, oil viscosity *μ* = 0.039 Pa·s, crown height *δ* = 4 μm, minimum oil film thickness *c* = 4 μm. As shown in Fig 5(b), the oil film pressure predicted by the present model is in good agreement with the result of Bertocchi et al.[41], as well as Gu et al. [28].

### Discussion on cavitation boundary conditions

At present, two well-known cavitation boundary conditions exist. The first one is Reynolds boundary condition, which only considers the oil film rupture and is much easier for implement in numerical calculation. The second one is the JFO boundary condition, which considers both the oil film rupture and reformulation, and it also meets the requirements of mass conservation. The JFO boundary condition can capture the features of cavitation more accurately, but it is difficult to implement in numerical calculation. In some cases, the Reynolds boundary condition can give quite similar results as compared with those given by JFO boundary condition, even though it does not consider the oil film reformulation. However, in the lubrication of a compression ring-cylinder liner system with micro dimples, the oil film cavitation is much more complex due to the time-varying load and sliding speed of the compression ring. In order to investigate the applicability of the two cavitation boundary conditions to the lubrication analysis of compression ring-cylinder liner system with micro spherical dimples on the liner, the minimum oil film thicknesses, average friction forces, and wear load are compared.

**Fig 6:**
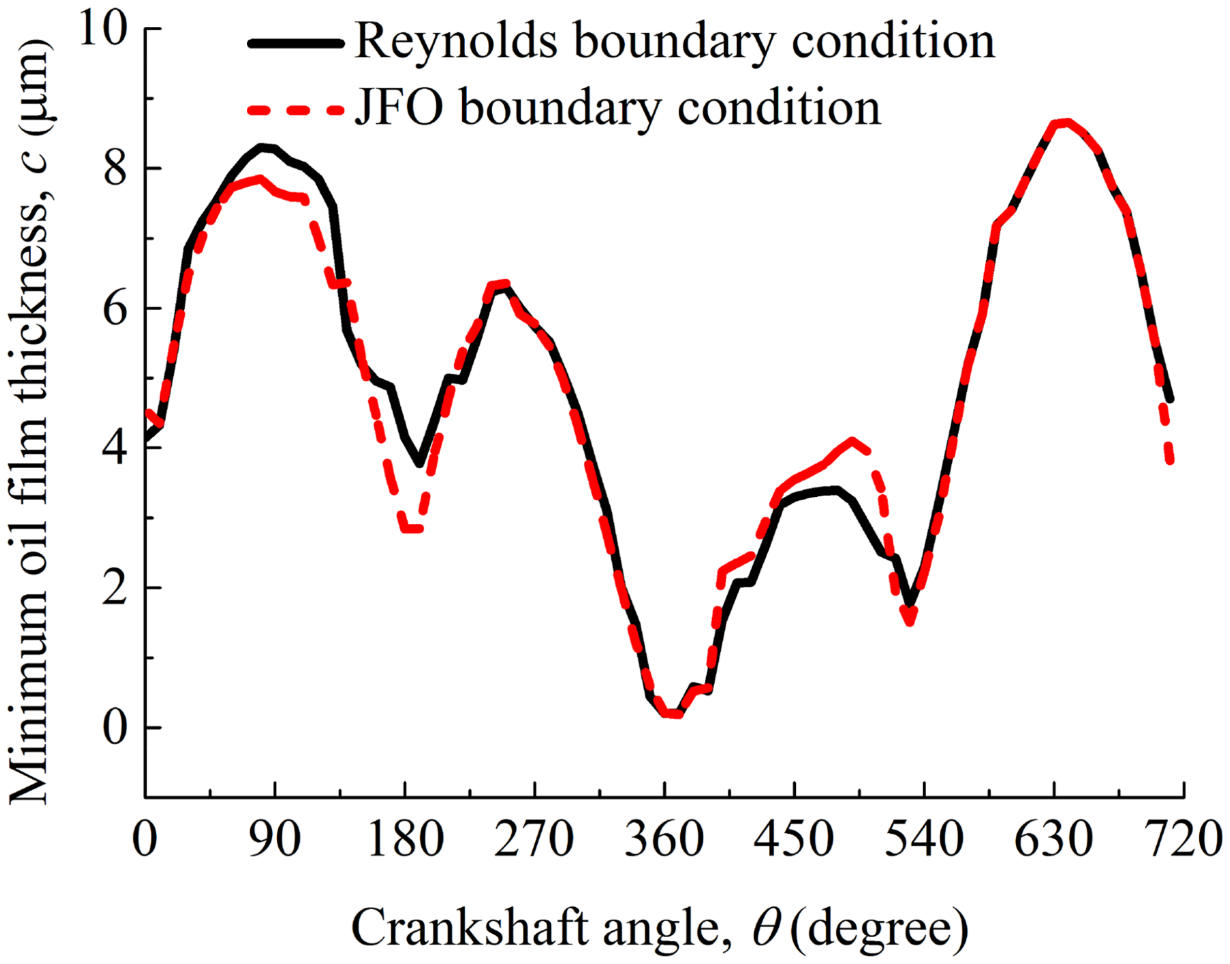
Minimum oil film thickness between the compression ring and the cylinder liner when JFO boundary condition and Reynolds boundary condition are used.

**Fig 7:**
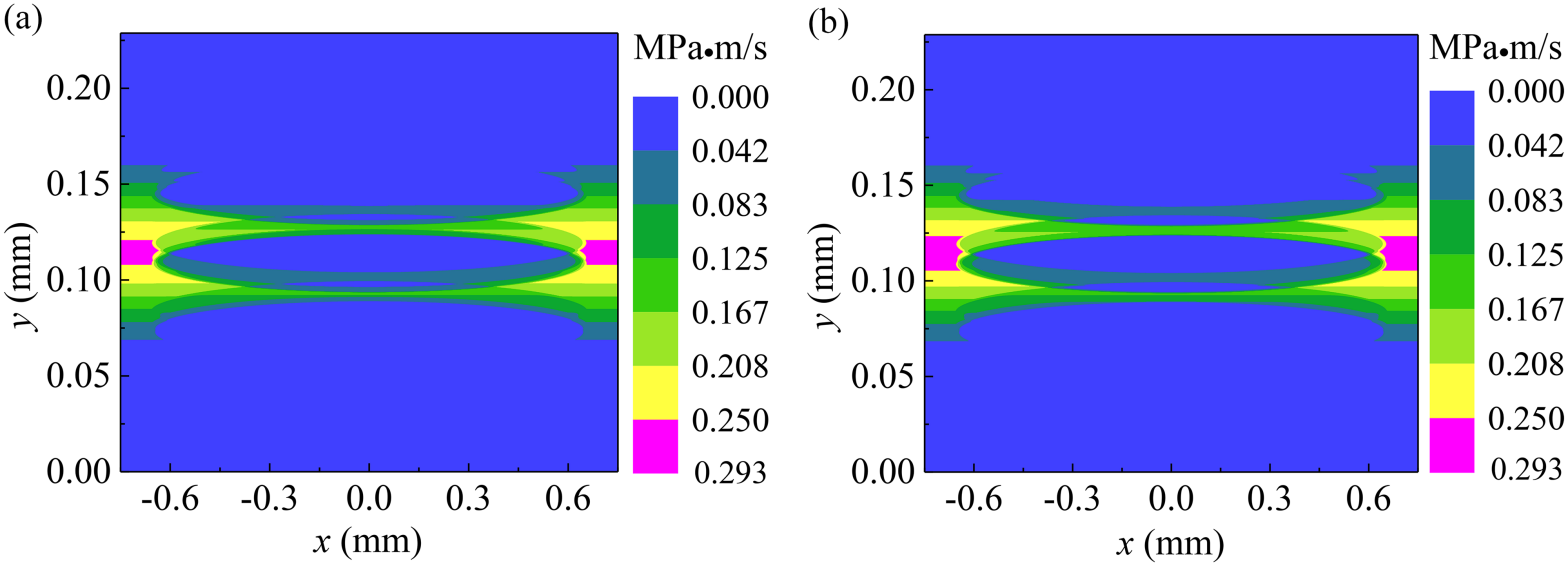
Wear load on the compression ring face when JFO boundary condition and Reynolds boundary condition are used. **(a)** wear load on the compression ring face when JFO boundary condition is used. **(b)** wear load on the compression ring face when Reynolds boundary condition is used.

When dimple area density *s* = 0.6, depth *h*_*d*_ = 7 μm, and radius *r*_*d*_ = 100 μm, Fig 6 shows the minimum oil film thickness between the compression ring and the cylinder liner when JFO boundary condition and Reynolds boundary condition are used, respectively. As shown in Fig 6, the cavitation boundary conditions have great influence on the minimum oil film thickness, especially near the middle of intake and power strokes (corresponding to *θ* = 90° and *θ* = 450°) and the bottom dead center (*θ* = 180°). Fig 7 shows the wear load on the compression ring face when JFO boundary condition and Reynolds boundary condition are used, respectively. As shown in Fig 7, the wear load on the compression ring face is overestimated when Reynolds boundary condition is used. Table 2 shows the average friction forces and their difference rates of the compression ring-cylinder liner system when JFO boundary condition and Reynolds boundary condition are used, respectively. What is found is that the cavitation boundary conditions have great influence on the average boundary friction force, and the average friction forces are overestimated when Reynolds boundary condition is used. Therefore, in order to predict the friction characteristics and minimum oil film thickness accurately, the oil film rupture and reformulation should be addressed in lubrication analysis of a textured compression ring-cylinder liner system.

**Table 2:** Average friction forces and their difference rates for the compression ring-cylinder liner system with dimples on the liner when JFO boundary condition and Reynolds boundary condition are used.

Parameter	JFO	Reynolds	Difference rate
**Average total friction force, *F* (N)**	11.38	11.55	1.49%
**Average hydrodynamic friction force, *F***_***v***_ **(N)**	8.75	8.79	0.51%
**Average boundary friction force, *F***_***b***_ **(N)**	2.63	2.76	4.94%

### Effect of dimple parameters on lubrication performance

In order to obtain the maximum minimum oil film thickness and minimum average friction force, an extensive parametric study is carried out to obtain the suitable dimple radius, area density, and depth. In the parametric study, the mass conservative JFO cavitation boundary condition is applied to the presented mixed lubrication model to address the oil film rupture and reformulation.

**Fig 8:**
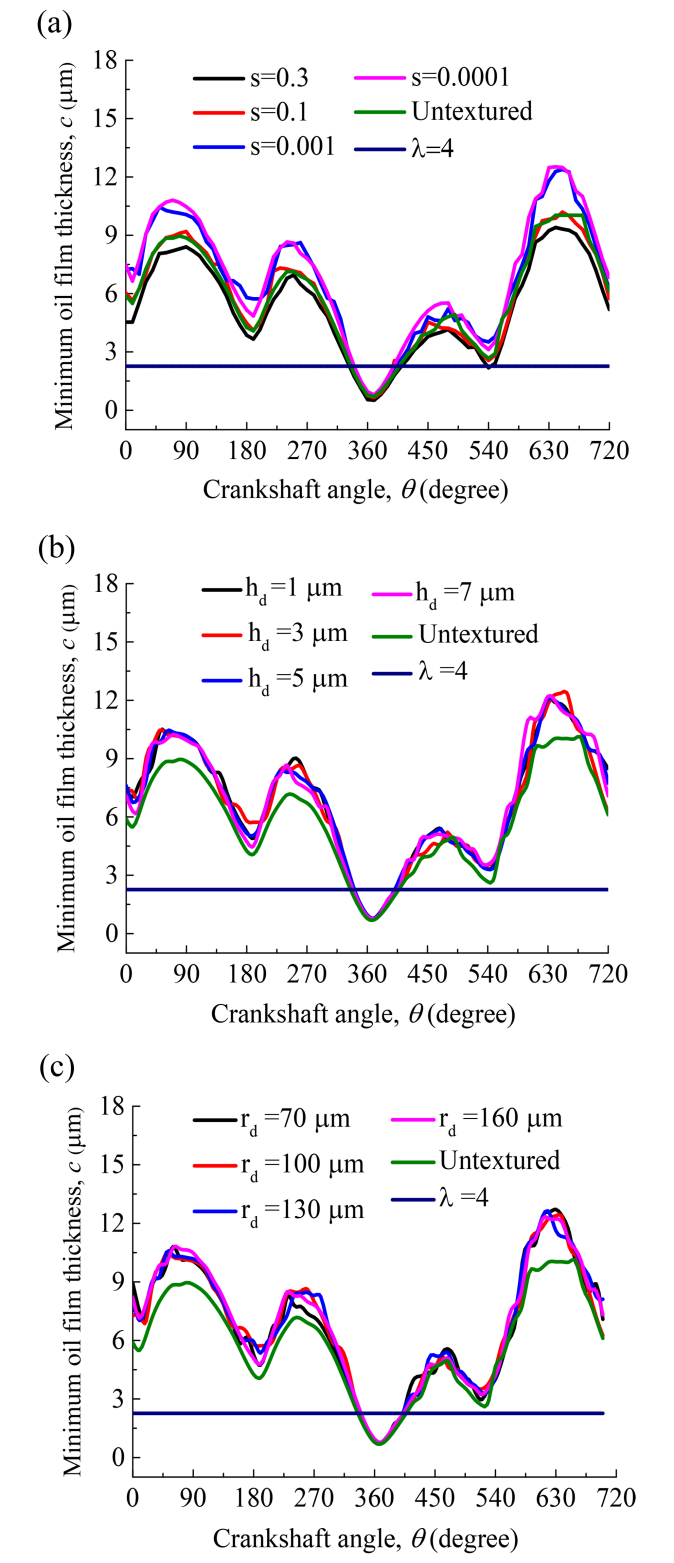
Minimum oil film thickness versus crankshaft angle for various dimple area densities, depths and radii. **(a)** minimum oil film thickness versus crankshaft angle for various dimple area densities. **(b)** minimum oil film thickness versus crankshaft angle for various dimple depths. **(c)** minimum oil film thickness versus crankshaft angle for various dimple radii.

**Fig 9:**
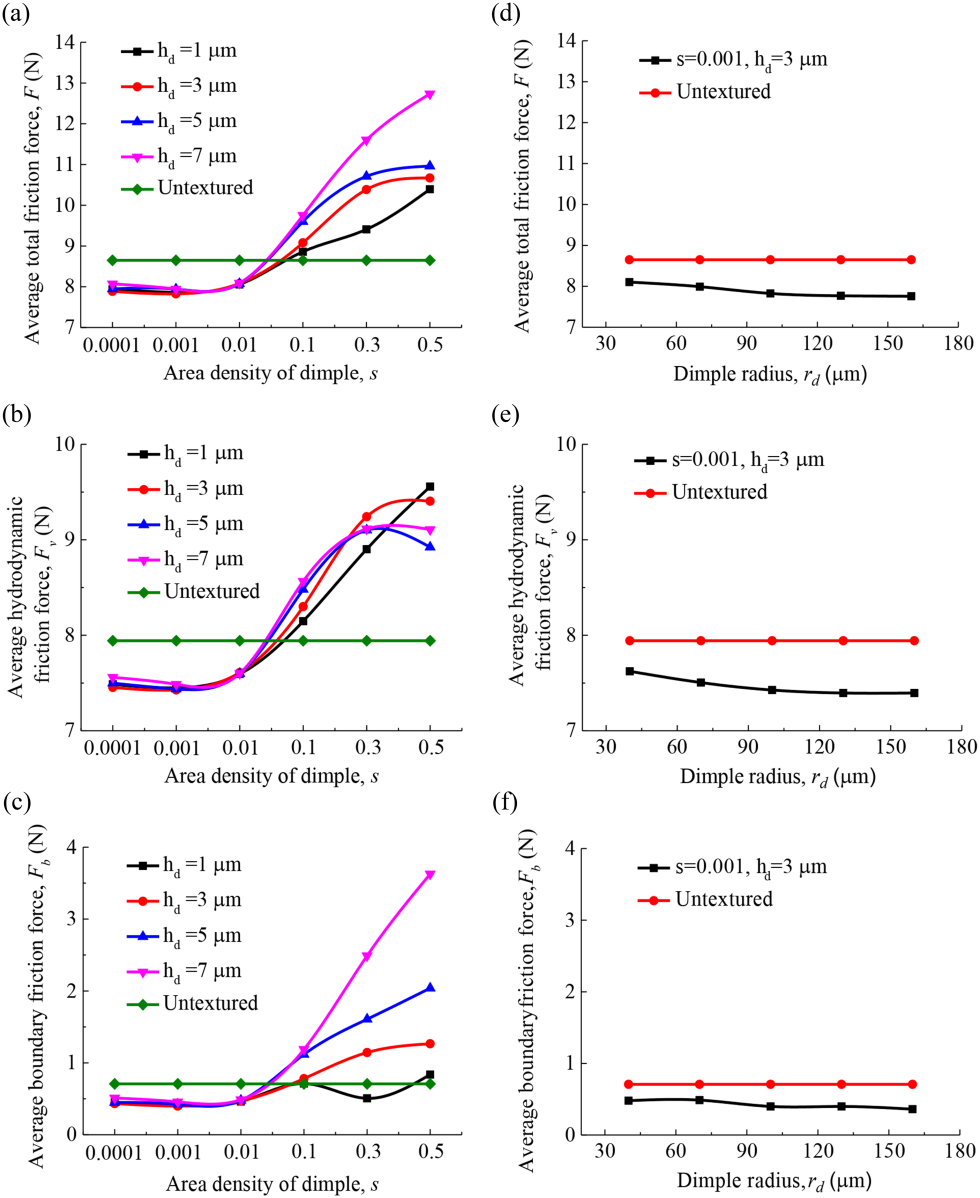
Effect of dimple area density, depth and radius on the average friction forces. **(a)** effect of dimple area density on the average total friction force for various dimple depths. **(b)** effect of dimple area density on the average hydrodynamic friction force for various dimple depths. **(c)** effect of dimple area density on the average boundary friction force for various dimple depths. **(d)** effect of dimple radius on the average total friction force. (e) effect of dimple radius on the average hydrodynamic friction force. **(f)** effect of dimple radius on the average boundary friction force.

The minimum oil film thickness versus crankshaft angle over one complete engine cycle for various dimple area densities is shown in Fig 8(a). As shown in Fig 8(a), the lubrication regime of the compression ring-cylinder liner system is changing from hydrodynamic lubrication (*λ*>4, corresponding minimum oil film thickness *c*>2.26 μm) to mixed lubrication (*λ*<4, corresponding minimum oil film thickness *c*<2.26 μm) when the compression ring reaches near the top dead center (*θ* = 360°). In the hydrodynamic lubrication region, what is found is that the dimple area density has a important impact on the minimum oil film thickness. In the mixed lubrication region, the influence of dimple area density on the minimum oil film thickness is limited. Compared with the untextured compression ring-cylinder liner system, the minimum oil film thickness in all strokes increases when smaller area density(*s*<0.1) is chosen, and a significant increase in the minimum oil film thickness is obtained when the compression ring reaches near the middle of the strokes. Fig 8(b) and 8(c) show the minimum oil film thickness versus crankshaft angle over one complete engine cycle for various dimple depths and radii when dimple area density *s* = 0.001. As shown in Fig 8(b) and 8(c), the dimple depth and radius have less effect on the minimum oil film thickness in comparison with the dimple area density. Fig 9(a)–9(c) show the average friction forces of the compression ring-cylinder liner system versus dimple area density for various dimple depths. As can be seen in Fig 9(a)–9(c), the average total friction force, average hydrodynamic friction force, and average boundary friction force all decrease at first and then increase slightly with the decrease of dimple area density. The dimples with smaller area density and depth can reduce the average total friction force, average hydrodynamic friction force, and average boundary friction force of compression ring-cylinder liner system greatly. When dimple area density *s* = 0.001 and depth *h*_*d*_ = 3 μm, the minimum average total friction force, average hydrodynamic friction force, and average boundary friction force can be obtained. Fig 9(d)–9(f) present the influence of dimple radius on the average friction forces for the dimples with area density *s* = 0.001 and depth *h*_*d*_ = 3 μm. What is observed is that the average total friction force, average hydrodynamic friction force, and average boundary friction force all decrease with the increase of dimple radius, but the decrease is very small. When dimple radius varies from 100 μm to 160 μm, the changes of average total friction force and average hydrodynamic friction force are both small, and such changes can be ignored. By comparison between the textured and untextured compression ring-cylinder liner systems, the average total friction force is reduced about 10.34%, the average hydrodynamic friction force is reduced about 6.89%, and the average boundary friction force is reduced about 48.98% when dimples area density *s* = 0.001, depth *h*_*d*_ = 3 μm, and radius *r*_*d*_ = 160 μm are chosen.

### Comparison of friction characteristics

**Fig 10:**
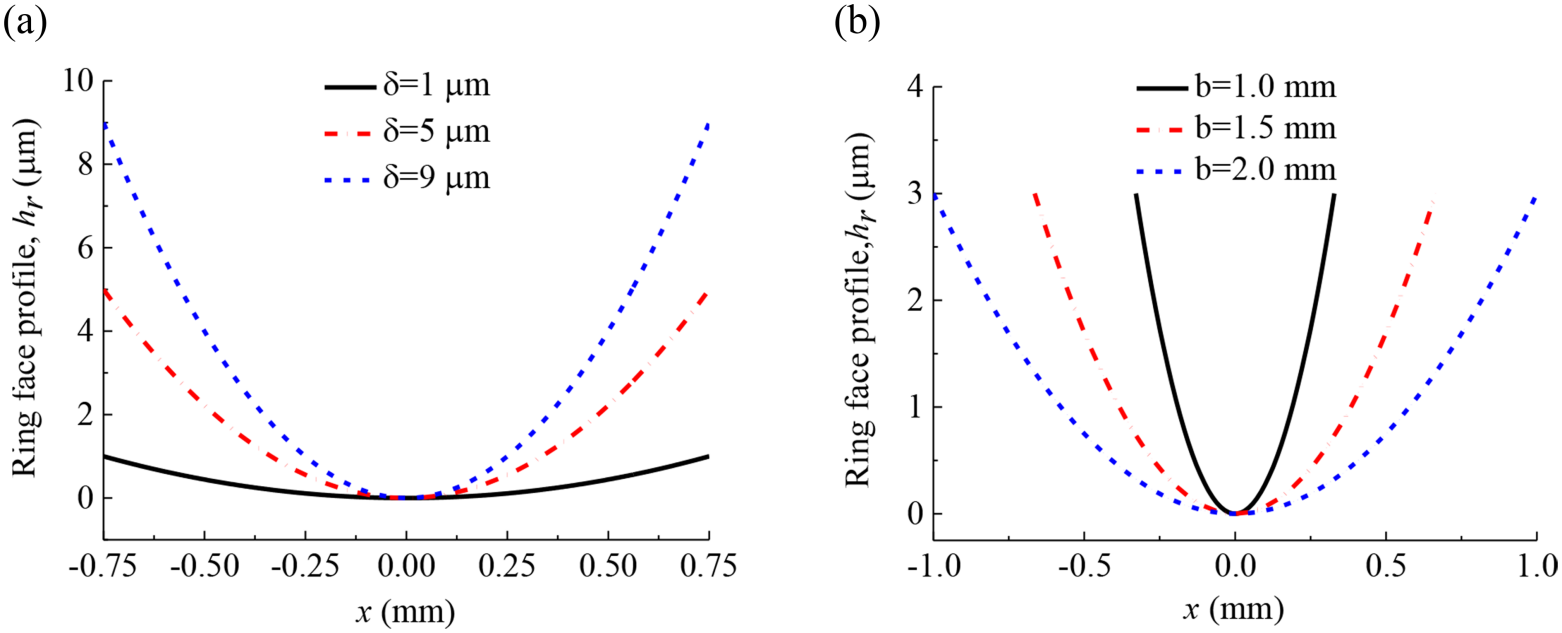
Face profiles of the compression ring with different crown heights and axial widths. **(a)** face profiles of the compression ring with crown height *δ* = 1 μm, 5 μm, 9 μm when axial width *b* = 1.5 mm. **(b)** face profiles of the compression ring with axial width *b* = 1.0 mm, *b* = 1.5 mm, *b* = 2.0 mm when crown height *δ* = 3 μm.

For the barrel-shaped compression ring-cylinder liner system, the face profile of compression ring (described by crown height and axial width) has a profound influence on the hydrodynamic pressure. The dimples textured on the liner have different friction reduction effects for different compression ring face profiles. The average friction forces, wear loads, and power losses of a compression ring-cylinder liner system with and without dimples are compared for the compression ring with different face profiles, respectively. In this study, six face profiles of compression ring are considered, as shown in Fig 10.

**Fig 11:**
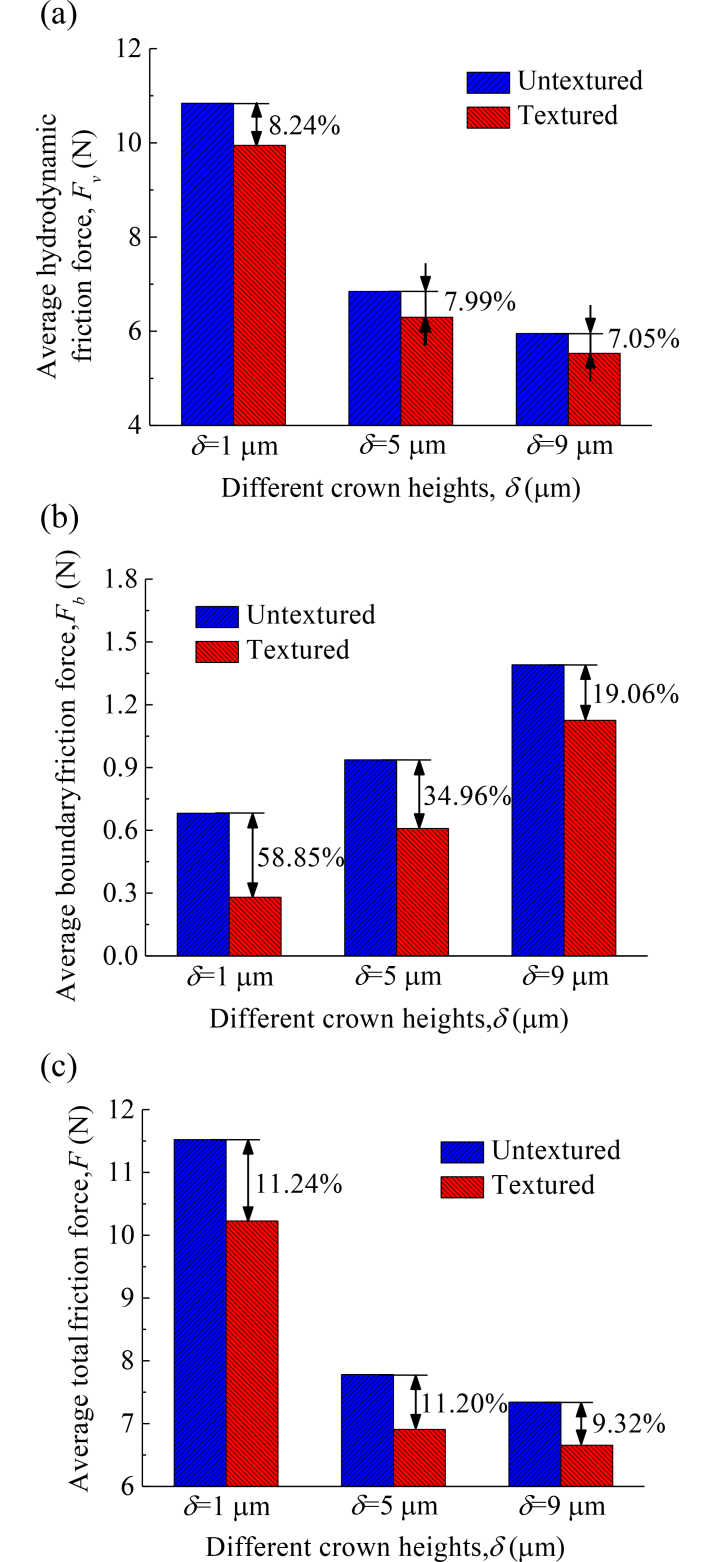
Average friction forces of the textured and untextured compression ring-cylinder liner systems for the ring with various crown heights. **(a)** average hydrodynamic friction force. **(b)** average boundary friction force. **(c)** average total friction force.

Fig 11 shows the average hydrodynamic friction forces, average boundary friction forces, and average total friction forces of the compression ring-cylinder liner system with and without dimples (dimple area density *s* = 0.001, depth *h*_*d*_ = 3 μm, and radius *r*_*d*_ = 160 μm) for crown heights *δ* = 1 μm, 5 μm, and 9 μm when axial width *b* = 1.5 mm. The reduction percentages of average friction forces with and without dimples are also shown in Fig 11. As shown in Fig 11(a) and 11(b), the reduction percentages of average hydrodynamic friction force and average boundary friction force are different for different crown heights. Higher reduction percentages of average hydrodynamic friction force and average boundary friction force are obtained when crown height is smaller (*δ* = 1 μm). Fig 11(c) shows the average total friction forces of the compression ring-cylinder liner system with and without dimples. Similar to the results of Fig 11(a) and 11(b), higher reduction percentage of average total friction force is also obtained when crown height is smaller (*δ* = 1 μm).

**Fig 12:**
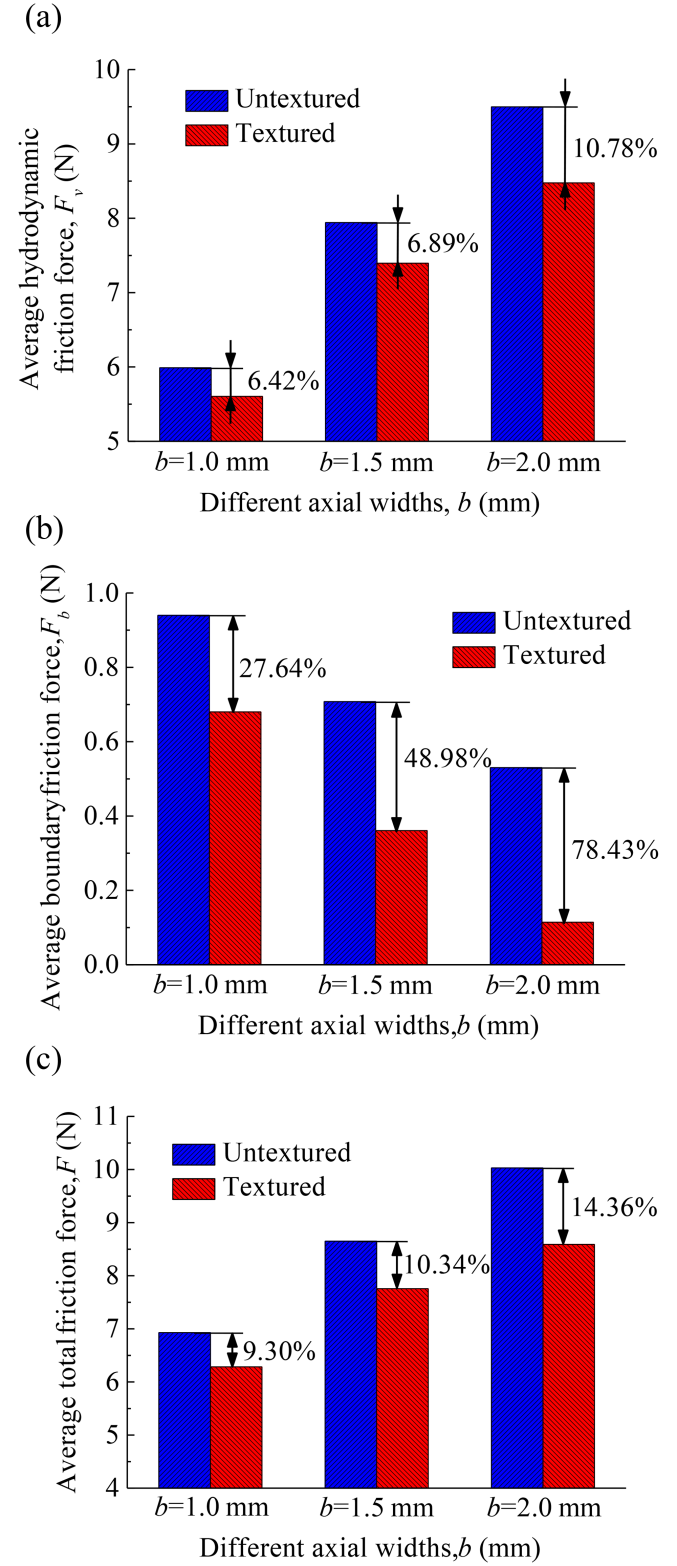
Average friction forces of the textured and untextured compression ring-cylinder liner systems for the ring with various axial widths. **(a)** average hydrodynamic friction force. **(b)** average boundary friction force. **(c)** average total friction force.

Fig 12 shows the average hydrodynamic friction forces, average boundary friction forces, and average total friction forces of the compression ring-cylinder liner system with and without dimples for axial widths *b* = 1.0 mm, 1.5 mm, and 2.0 mm when crown height *δ* = 3 μm. As shown in Fig 12, the reduction percentages of average hydrodynamic friction force, average boundary friction force, and average total friction force are different for different axial widths. Higher reduction percentages of average hydrodynamic friction force, average boundary friction force, and average total friction force are obtained when axial width is larger (*b* = 2.0 mm). From Figs 11 and 12, what is observed is that the reduction percentages of average boundary friction force are larger than average hydrodynamic friction force. Therefore, the dimples textured on the liner can produce a larger reduction of friction in the mixed lubrication region because the boundary friction force accounts for a large proportion of total friction force when the textured compression ring reaches near the top dead center (i.e., mixed lubrication regime).

**Fig 13:**
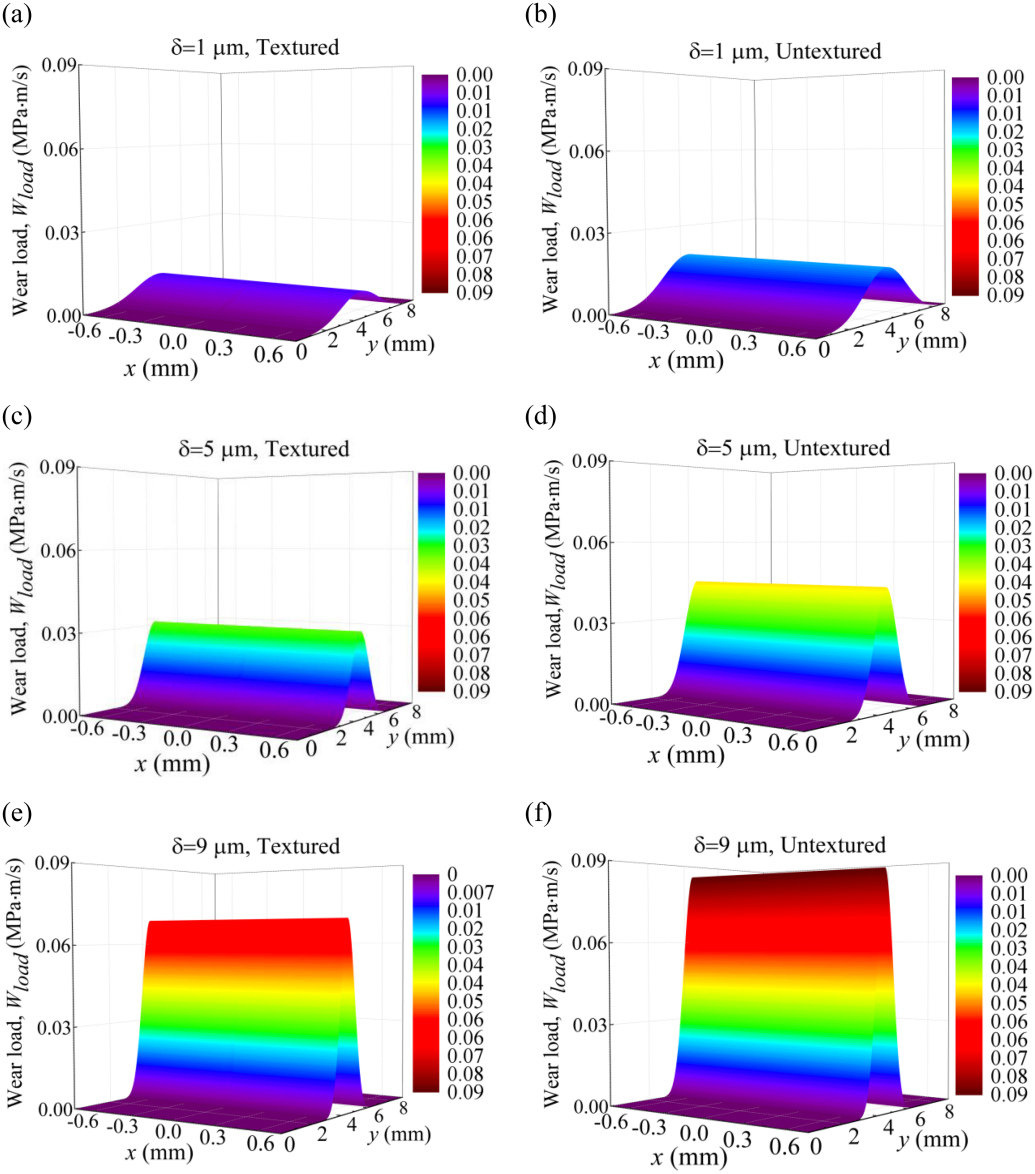
Wear loads of the textured and untextured compression ring-cylinder liner systems. **(a)** wear load of the textured compression ring-cylinder liner system for crown height *δ* = 1 μm. **(b)** wear load of the untextured compression ring-cylinder liner system for crown height *δ* = 1 μm. **(c)** wear load of the textured compression ring-cylinder liner system for crown height *δ* = 5 μm. **(d)** wear load of the untextured compression ring-cylinder liner system for crown height *δ* = 5 μm. **(e)** wear load of the textured compression ring-cylinder liner system for crown height *δ* = 9 μm. **(f)** wear load of the untextured compression ring-cylinder liner system for crown height *δ* = 9 μm.

**Fig 14:**
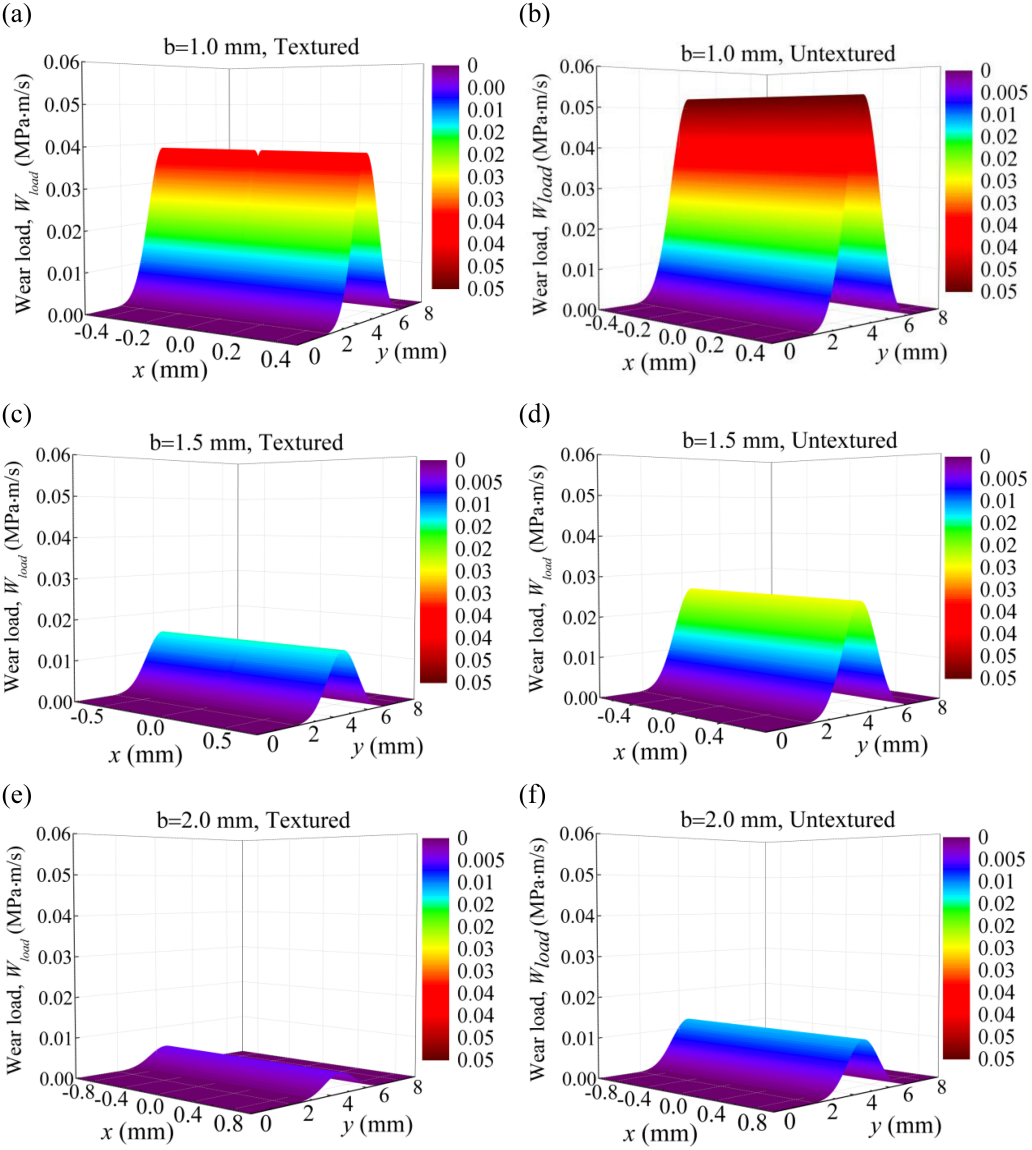
Wear loads of the textured and untextured compression ring-cylinder liner systems. **(a)** wear load of the textured compression ring-cylinder liner system for axial width *b* = 1.0 mm. **(b)** wear load of the untextured compression ring-cylinder liner system for axial width *b* = 1.0 mm. **(c)** wear load of the textured compression ring-cylinder liner system for axial width *b* = 1.5 μm. **(d)** wear load of the untextured compression ring-cylinder liner system for axial width *b* = 1.5 μm. **(e)** wear load of the textured compression ring-cylinder liner system for axial width *b* = 2.0 μm. **(f)** wear load of the untextured compression ring-cylinder liner system for axial width *b* = 2.0 μm.

Fig 13 shows the wear loads on the compression ring with and without dimples on the liner for crown heights *δ* = 1 μm, 5 μm, and 9 μm when axial width *b* = 1.5 mm. As shown in Fig 13, with the increase of crown height, the maximum wear load is increased and the area of the wear region is decreased for both the textured and untextured liners. By comparison between the textured and untextured compression ring-cylinder liner systems, lower wear load is obtained when micro dimples are textured on the liner, and the maximum wear loads are reduced about 47.97%, 29.28%, and 19.30% for crown heights *δ* = 1 μm, 5 μm, and 9 μm. Fig 14 shows the wear loads on the compression ring with and without dimples on the liner for axial widths *b* = 1.0 mm, 1.5 mm and 2.0 mm when crown height *δ* = 3 μm. As shown in Fig 14, the maximum wear loads decrease with the increase of the axial width for both the textured and untextured liners. By comparison between the textured and untextured compression ring-cylinder liner systems, the dimples textured on the liner reduce the wear load of compression ring significantly, and the maximum wear loads are reduced about 25.86%, 42.05%, and 60.81% for axial widths *b* = 1.0 mm, 1.5 mm, and 2.0 mm. The numerical results indicate that the dimples textured on the liner can reduce the wear on the compression ring effectively, especially for the compression ring with smaller crown height or larger axial width.

**Fig 15:**
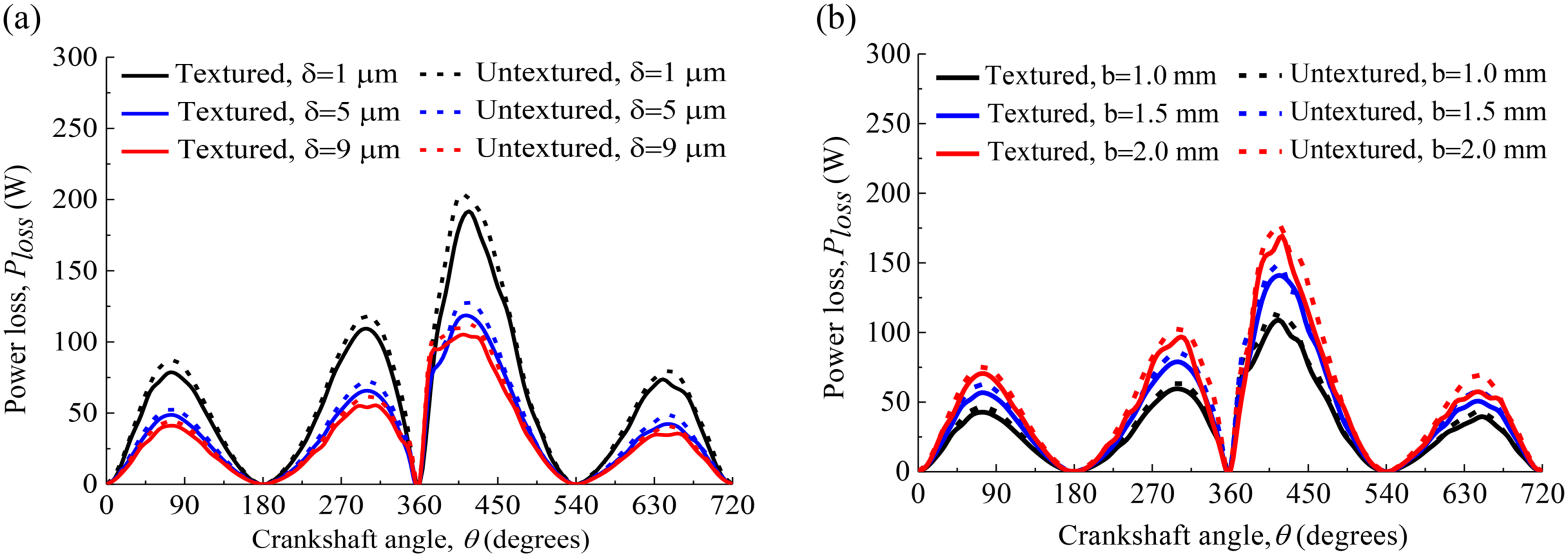
Power loss of the textured and untextured compression ring-cylinder liner systems. **(a)** crown heights *δ* = 1 μm, 5 μm and 9 μm when axial width *b* = 1.5 mm. **(b)** axial widths *b* = 1.0 mm, 1.5 mm and 2.0 mm when crown height *δ* = 3 μm.

Fig 15(a) shows the power losses of the compression ring-cylinder liner system with and without dimples on the liner for crown heights *δ* = 1 μm, 5 μm, and 9 μm when axial width *b* = 1.5 mm. Fig 15(b) shows the power losses of the compression ring-cylinder liner system with and without dimples on the liner for axial widths *b* = 1.0 mm, 1.5 mm, and 2.0 mm when crown height *δ* = 3 μm. From Fig 15, what is observed is that the power loss is significantly reduced after texturing when the compression ring reaches near the middle of the strokes. However, when the compression ring reaches near the top and bottom dead centers, the difference of power loss between the textured and untextured compression ring-cylinder liner systems is negligibly small because the sliding velocity of the compression ring is low. Moreover, it can also be seen that the power loss reduction is less affected by the crown height and axial width of the compression ring.

## Conclusions

By considering the surface roughness of the compression ring and cylinder liner, the average Reynolds equation and Greenwood asperity contact model are employed to investigate the effects of liner dimples on the lubrication performance of compression ring-cylinder liner system under engine-like conditions. The mass conservation JFO boundary condition is adopted to determine the rupture and reformulation positions of fluid film. The related conclusions can be made as follows:

The area density of spherical dimple has an important impact on the average friction forces and minimum oil film thickness. Smaller dimple area density can increase the minimum oil film thickness and decrease the average friction forces significantly. The radius of spherical dimple has less effect on the minimum oil film thickness and average friction forces in comparison with the dimple area density. The minimum average friction forces and maximum minimum oil film thickness can be obtained for the spherical dimples with suitable area density and depth.

The spherical dimples textured on the cylinder liner can produce a larger reduction of friction in mixed lubrication region than in hydrodynamic lubrication region. Higher reduction percentages of average friction forces and wear load can be obtained when the compression ring crown height is smaller or axial width is larger. The power loss is significantly reduced after texturing when the compression ring reaches near the middle of the strokes. The compression ring crown height and axial width have less effect on the power loss reduction.

## References

[1] Becker EP. Trends in tribological materials and engine technology. Tribology International. 2004; 37(7): 569–575.

[2] Ronen A, Etsion I, Kligerman Y. Friction-reducing surface-texturing in reciprocating automotive components. Tribology Transaction. 2001; 44(3): 359–366.

[3] Nakano M, Korenaga A, Korenaga A, Miyake K, Murakami T, Ando Y, et al Applying micro-texture to cast iron surfaces to reduce the friction coefficient under lubricated conditions. Tribology Letters. 2007; 28(2): 131–137.

[4] Ramesh A, Akram W, Mishra SP, Cannon AH, Polycarpou AA, King WP. Friction characteristics of micro textured surfaces under mixed and hydrodynamic lubrication. Tribology International. 2013; 57: 170–176.

[5] Biboulet N, Bouassida H, Lubrecht AA. Cross hatched texture influence on the load carrying capacity of oil control rings. Tribology International. 2015; 82: 12–19.

[6] Costa HL, Hutchings IM. Hydrodynamic lubrication of textured steel surfaces under reciprocating sliding conditions. Tribology International. 2007; 40(8): 1227–1238.

[7] Gropper D, Wang N, Harvey TJ. Hydrodynamic lubrication of textured surfaces: A review of modeling techniques and key findings. Tribology International. 2016; 94: 509–529.

[8] Etsion I. State of the art in laser surface texturing. ASME Journal of Tribology. 2005; 127(1): 248–253.

[9] Johansson S, Nilsson PH, Ohlsson R, Anderberg C, Rosén BG. New cylinder liner surfaces for low oil consumption. Tribology International. 2008; 41(9): 854–859.

[10] Gu CX, Meng XH, Xie YB, Kong XL. Performance of surface texturing during start up under starved and mixed lubrication. ASME Journal of Tribology. 2016; 139(1): 011702-1-11.

[11] Kligerman Y, Etsion I, Shinkarenko A. Improving tribological performance of piston rings by partial surface texturing. ASME Journal of Tribology. 2005; 127(3): 632–638.

[12] Etsion I, Sher E. Improving fuel efficiency with laser surface textured piston rings. Tribology International. 2009; 42(4): 542–547.

[13] Gadeschi GB, Backhaus K, Knoll G. Numerical analysis of laser-textured piston-rings in the hydrodynamic lubrication regime. ASME Journal of Tribology. 2012; 134(4): 041702-1-8.

[14] Zavos AB, Nikolakopoulos PG. Simulation of piston ring tribology with surface texturing for internal combustion engines. Lubrication Science. 2015; 27(3): 151–176.

[15] Shen C, Khonsari MM. The effect of laser machined pockets on the lubrication of piston ring prototypes. Tribology International. 2016; 101: 273–283.

[16] Usman A, Park CW. Optimization the tribological performance of textured piston ring-liner contact for reduced friction losses in SI engine: warm operating conditions. Tribology International. 2016; 99: 224–236.

[17] Tomanik E. Modelling the hydrodynamic support of cylinder bore and piston rings with laser textured surfaces. Tribology International. 2013; 59: 90–96.

[18] Yin BF, Li XD, Fu YH, Yun W. Effect of laser textured dimples on the lubrication performance of cylinder liner in diesel engine. Lubrication Science. 2012; 24(7): 293–312.

[19] Zhou YK, Zhu H, Tang W, Ma CB, Zhang WQ. Development of the theoretical model for the optimal design of surface texturing on cylinder liner. Tribology International. 2012; 52: 1–6.

[20] Mohamad SA, Lu XQ, Zheng Q. Numerical modeling of lubrication of piston ring of two-stroke marine diesel engine considering the effect of multi-scale grooves on the cylinder liner. Proceedings of the Institution of Mechanical Engineers, Part J: Journal of Engineering Tribology. 2015; 229(8): 989–1002.

[21] Mohamad SA, Lu XQ, Zheng Q. Effect of cylinder liner oil grooves shape on two-stroke marine diesel engine’s piston ring friction force. Advances in Mechanical Engineering. 2015; 7(2): 837960.

[22] Smith EH. Optimising the design of a piston-ring pack using DoE methods. Tribology International. 2011; 44(1): 29–41.

[23] Zhan J, Yang MJ. The effects of dimple distribution angle on the tribology performance of a laser surface textured cylinder piston ring system. Lasers in Engineering. 2014; 29(1): 123–131.

[24] Gu CX, Meng XH, Xie YB, Yang YM. Effects of surface texturing on ring/liner friction under starved lubrication. Tribology International. 2016; 94: 591–605.

[25] Morris N, Rahmani R, Rahnejat H, King PD, Howell-Smith S. A numerical model to study the role of surface textures at top dead center reversal in the piston ring to cylinder liner contact. ASME Journal of Tribology. 2016; 138(2): 021703-1-11.

[26] Shahmohamadi H, Mohammadpour M, Rahmani R, Rahnejat H, Garner CP, Howell-Smith S. On the boundary conditions in Multi-phase flow through the piston ring-cylinder liner conjunction. Tribology International. 2015; 90: 164–174.

[27] Ausas RF, Jai M, Ciuperca IS, Buscaglia GC. Conservative one-dimensional finite volume discretization of a new cavitation model for piston-ring lubrication. Tribology International. 2013; 57: 54–66.

[28] Gu CX, Meng XH, Xie YB, Zhang D. Mixed lubrication problems in the presence of textures: An efficient solution to the cavitation problem with consideration of roughness effects. Tribology International. 2016; 103: 516–528.

[29] Zhang JY, Meng YG. Direct observation of cavitation phenomenon and hydrodynamic lubrication analysis of textured surfaces. Tribology Letters. 2012; 46(2): 147–158.

[30] Checo HM, Ausas RF, Jai M, Cadalen JP, Choukroun F, Buscaglia GC. Moving textures: simulation of a ring sliding on a textured liner. Tribology International. 2014; 72: 131–142.

[31] Patir N, Cheng HS. An average flow model for determining effects of three-dimensional roughness on partial hydrodynamic lubrication. Journal of Lubrication Technology. 1978; 100(1): 12–17.

[32] Wu C, Zheng L. An average Reynolds equation for partial film lubrication with a contact factor. ASME Journal of Tribology. 1989; 111(1): 220–229.

[33] Wang YS, Wang QJ, Lin C. Mixed lubrication of coupled journal-thrust-bearing systems including mass conserving cavitation. ASME Journal of Tribology. 2003; 125(4): 747–755.

[34] Xiong SW, Wang QJ. Steady-state hydrodynamic lubrication modeled with the Payvar-Salant mass conservation model. ASME Journal of Tribology. 2012; 134(3): 031703-1-16.

[35] Meng XH, Gu CX, Xie YB, Li WX. A two-dimensional starved lubrication analysis method for textured surfaces. International Journal of Engine Research. 2016; 17(10): 1062–1076.

[36] Greenwood JA, Tripp JH. The contact of two nominally flat rough surfaces. Proceedings of the Institution of Mechanical Engineers. 1970; 185(1): 625–633.

[37] Gu CX, Meng XH, Xie YB, Fan JZ. A thermal mixed lubrication model to study the textured ring/liner conjunction. Tribology International. 2016; 101: 178–793.

[38] Morris N, Rahmani R, Rahnejat H, King PD, Fitsimons B. Tribology of piston compression ring conjunction under transient thermal mixed regime of lubrication. Tribology International. 2013; 59: 248–258.

[39] Kligerman Y, Shinkarenko A. Analysis of friction in surface textured components of reciprocating mechanism. Proceedings of the Institution of Mechanical Engineers, Part J: Journal of Engineering Tribology. 2015; 229(4): 336–349.

[40] Gulwadi SD. Analysis of tribological performance of a piston ring pack. ASME Journal of Tribology. 2000; 43(2): 151–162.

[41] Bertocchi L, Dini D, Giacopini M, Fowell MT, Baldini A. Fluid film lubrication in the presence of cavitation: a mass-conserving two-dimensional formulation for compressible, piezoviscous and non-Newtonian fluids. Tribology International. 2013; 67: 61–71.

